# A structured dataset of urban climate actions in European cities

**DOI:** 10.1016/j.dib.2025.112311

**Published:** 2025-11-27

**Authors:** Iskander Ben Rjiba, Georgina Tóth-Nagy, Ágnes Rostási, Petra Gyurácz-Németh, Viktor Sebestyén

**Affiliations:** a Sustainability Solutions Research Lab, University of Pannonia, Egyetem str. 10, Veszprém, H-8200, Hungary; b Research Institute of Biomolecular and Chemical Engineering, University of Pannonia, Egyetem str. 10, Veszprém, H-11 8200, Hungary; c Department of Tourism, University of Pannonia, Egyetem str. 10, Veszprém, H-8200, Hungary

**Keywords:** Climate action planning, Urban sustainability, Structured dataset, Decision-tree modeling, Geodemographic features, Spatial indicators, Covenant of mayors, Adaptation strategies

## Abstract

Urban climate planning requires structured datasets to support predictive modeling. This article presents a dataset based mainly on 454 climate action reports published by 443 European cities, available on the Covenant of Mayors platform. It includes 24 features classified into geodemographic, action-related, and spatial distribution categories. In addition, data were sourced from municipal reports, public databases (e.g., ODP, Weather Spark), and geospatial APIs. The structured dataset covers a wide geographic distribution in Europe and reflects the scope of adaptation-oriented climate action plans. It is built upon the analysis and evaluation of actions already implemented by cities, enabling consistent classification of adaptation strategies and supports climate actions modeling with improved accuracy, offering an interesting tool for data-driven climate action planning.

Specifications Table

**Table:** 

Subject	Earth & Environmental Sciences
Specific subject area	Climate Action Planning and Urban Modeling.
Type of data	Tables, Figures, xlsx file Raw, Processed, Filtered.
Data collection	Data were collected from 454 climate action plans published by 443 cities on the Covenant of Mayors website. Action features were manually extracted and categorized using text analysis. Geodemographic data were sourced from public datasets (ODP, Weather Spark). Spatial features were retrieved using Python scripts and OpenStreetMap APIs.
Data source location	443 cities located in Europe
Data accessibility	Repository name: Database-of-article-How-should-climate-actions-be-planned-Model-lessons-from-published-action-plans Data identification number: 2437,596 Direct URL to data: https://github.com/Iskander523/Database-of-article-How-should-climate-actions-be-planned-Model-lessons-from-published-action-plans-.git
Related research article	Ben Rjiba, I., Tóth-Nagy, G., Rostási, Á., Gyurácz-Németh, P., Sebestyén, V. [3]. How should climate actions be planned? Model lessons from published action plans. [Journal of Environmental Management, Volume 370, November 2024, 122,648. [3]

## 1 Value of the Data

• The dataset supports evidence-based climate action planning by providing structured information on 454 real-world climate action plans submitted by 443 cities under the Covenant of Mayors initiative.
• The data can be reused by researchers to build and test machine learning models for classifying urban climate strategies based on city-specific characteristics such as population, climate class, energy use, and spatial distribution.
• The dataset enables comparative analysis across cities by standardizing the key features selected into different categories.
• Urban planners and policy analysts can use this dataset to assess trends in climate adaptation and mitigation strategies, helping prioritize interventions in similar urban contexts.
• This is one of the first publicly structured datasets linking published city climate actions with spatial and demographic variables in a model-ready format, with a specific focus on emissions reduction targets (2030 and 2050), allowing evaluation of the efficiency and impact of planned actions on reduction percentages.
• The dataset is designed to derive adaptation action categories through the evaluation of actions already implemented, making it both a compilation of existing climate action plans and a basis for model creation.

## 2 Background

Previous compilations and comparisons of Climate Action Plans (CAPs) have addressed different aspects of urban climate governance. Page et al. highlighted the influence of cultural similarity and climate leadership on policy diffusion [8]. Bassett and Shandas evaluated the content of 20 CAPs using a structured scoring matrix [2], while Boswell et al. conducted a detailed content analysis of 30 plans, identifying key trends and typologies [4]. Hale et al. proposed a framework for assessing the progress and impact of subnational climate actions [6], and Pan et al. applied a comparative socio-ecological modeling approach to identify improvement opportunities in CAPs from Chicago and Stockholm [5]. Building on these contributions, the present work develops a structured, model-ready dataset of 454 climate action plans from 443 European cities, sourced from the Covenant of Mayors platform. It expands the geographical coverage and integrates geodemographic, spatial, and action-related categories to evaluate all major adaptation actions derived from already implemented measures.

The motivation behind creating this dataset was to address the growing need for structured, comparable data to support climate action planning at the city level. While thousands of municipal action plans have been published under the Covenant of Mayors initiative [1], these reports are unstructured and vary significantly in scope and terminology [11]. To enable consistent analysis and predictive modeling, data were systematically extracted from 454 published reports and categorized into geodemographic, spatial, and action-related feature groups, drawing on methodological approaches from climate adaptation and urban systems literature [9,10].

This dataset therefore represents a significant step beyond earlier CAP compilations by transforming fragmented reports into a harmonized and model-ready structure specifically designed for evaluating adaptation actions in European cities. It provides a unified basis for assessing the relationships between city characteristics and their implemented actions, allowing both comparative analysis and predictive applications.

This dataset can be used for building several model types related to climate action planning. In this specific case, it supports the methodology presented in the related research article titled *“How should climate actions be planned? Model lessons from published action plans.”* That study applies decision-tree modeling to identify climate actions suited to different urban contexts [7]. The dataset published provides the foundation for that framework and can serve broader applications in urban sustainability research.

## 3 Data Description

The dataset includes structured climate action information for 443 cities that submitted climate action plans to the Covenant of Mayors platform. The aim of the selection was to cover as many European countries as possible in order to capture greater variability and make differences in strategies between countries more visible. A total of 454 climate action reports were selected from 8747 reports available on the website, representing 5.2 % of the total. The data were collected and processed in 2024. Each city is represented by 24 features grouped into three categories: geodemographic (8 features), action-related (8 features), and spatial distribution indicators (8 features).

The features were selected precisely, in order to characterize the social and climatic profile of each city. Spatial distribution features describe land use and infrastructure, including total city area, landfill and industrial zones, agricultural and green areas, lake/water body coverage, cumulative building area, and total road length. These spatial indicators were collected using **Python** and the **OSMnx** library to access OpenStreetMap infrastructure data. Key functions included ‘graph_from_place ()’ and ‘geometries_from_place ()’.Missing values were imputed using means from geographically similar cities when applicable. This method was applied primarily to spatial distribution features (4 features). The feature with the highest proportion of missing data was the landfill area, with missing values for 152 cities, representing 33.48 % of the entire dataset. In contrast, the remaining features showed relatively limited data gaps: industrial area was missing for 58 cities (12.77 %), agricultural area for 74 cities (16.3 %), and lakes and water bodies area for 31 cities (6.8 %).

Data for variables like temperature and precipitation are extracted from recent available sources (2024) and represent average annual values. These Geodemographic and climate data were collected from publicly available databases, including Our Data Platform (ODP) and Weather Spark. The third category, action-related features, was extracted directly from the 454 climate action reports analyzed. The date of publication of these reports are variable from 2010 to 2024. It includes indicators such as the existence of adaptation plans, public awareness actions, GHG emissions per capita, emission reduction targets for 2030 and 2050, energy supply strategies, renewable energy ratios, and adaptation plan budgets (Table 1)

**Table 1:** Feature selection and classification.

Geodemographic features	Spatial distribution features	Action-related features
Population (capita)	Total city areas (km^2^)	Adaptation plans
Population density (capita \km^2^)	Landfill areas (km^2^)	Public awareness actions
Average annual Temperature ( °C)	Industrial areas (km^2^)	GHG emissions/capita (CO_2eq._\capita)
Average annual Precipitation (mm)	Agricultural areas (km^2^)	Emission reduction target in 2030 ( %)
Climate class	Lake and water body areas (km^2^)	Emission reduction target in 2050 ( %)
Climate hazards	Green areas (km^2^)	Energy supply (MWh)
HDI	Cumulative building area (km^2^)	Renewable energy ratio ( %)
Geographic location (Coastal/landlocked)	Total length of roads (km)	Budget of adaptation plans (Euro)

The dataset is stored in a single excel file titled ‘Article database (input).xlsx’, containing both raw and processed values. Missing values in selected features were imputed using the average values of geographically proximate cities, and certain non-impactful features were excluded during preprocessing.

## 4 Experimental Design, Materials and Methods

**Fig. 1:**
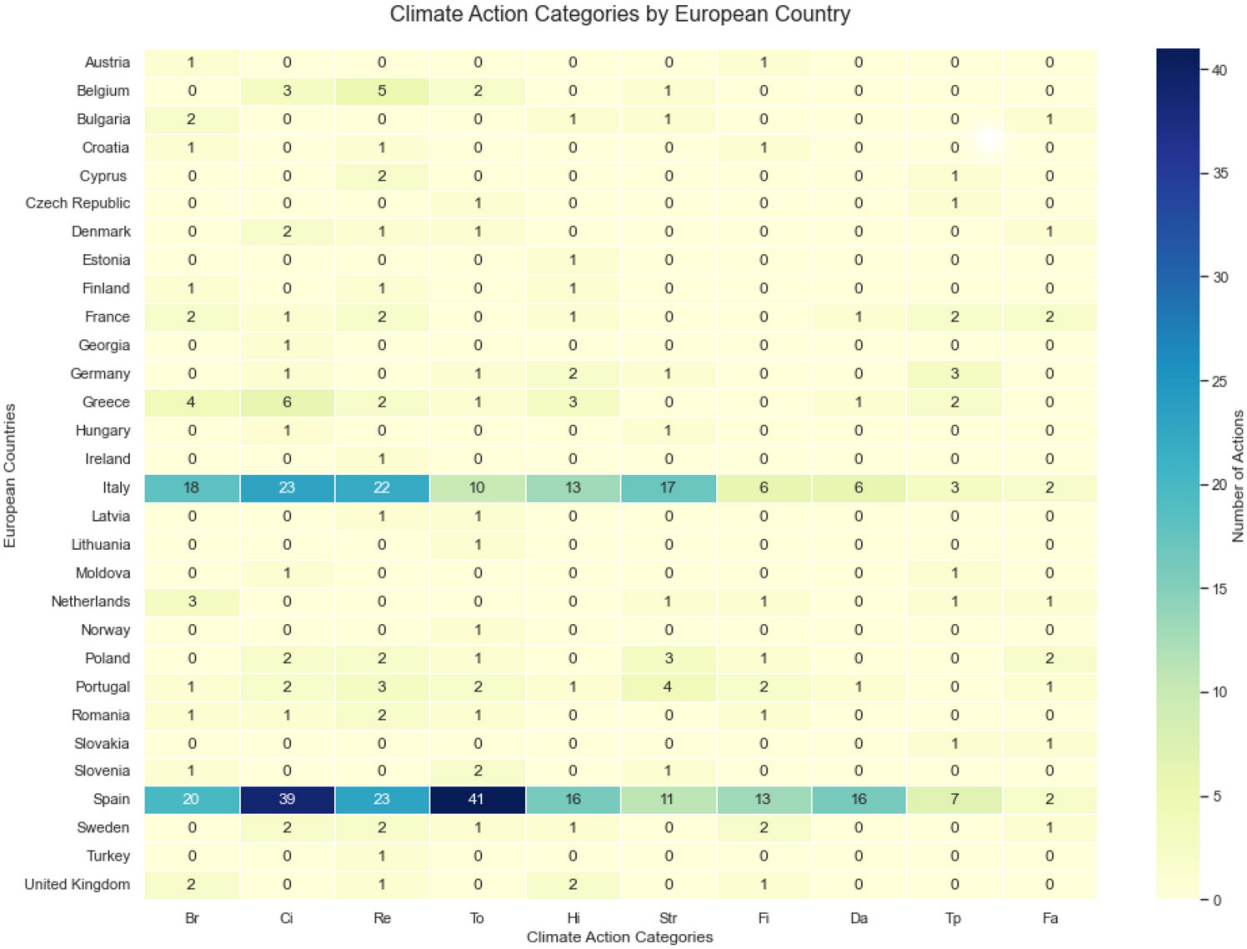
Distribution of climate action category in the different European countries.

The database was constructed through a multi-step methodology: After identifying and compiling 454 local climate action plans from 443 cities, a text mining approach was applied to analyze the descriptions of actions within these reports: The process involved extracting and analyzing the textual content of the different reports using keyword frequency and pattern recognition techniques. Specific terms related to urban climate strategies, such as energy efficiency, building renovation, flood prevention, green spaces, mobility, waste management, renewable energy, awareness raising, governance, monitoring and evaluation, were used to identify and group similar actions into main categories. Based on these findings, 10 climate action categories were derived to capture the diversity and commonalities of city-level responses (Fig. 1). Alongside this, additional data (geodemographic and spatial) was collected from external sources to complement the action-specific variables and enable structured comparative analysis throughout the different cases.

During the creation of the database, cities were selected to represent a wide range of scales from major metropolitan areas like Paris to small towns and rural villages such as Aldehuela de Liestos. This diversity in city size, geography, and administrative capacity is reflected in the range of values observed across key features. For example, the action plan budget varies from just 327.8 euro in Adamuz (a small municipality) to 300 million euro in Paris, highlighting the significant variation in financial capacity. Population density also varies dramatically, from 1.3 in Aldehuela de Liestos to 20,623.1 inhabitants/km² in Paris, one of Europe’s most densely populated cities. In terms of climate, average annual precipitation ranges from 238 mm in Almáchar, located in a semi-arid region of southern Spain, to 2923 mm in A Baña, situated in the rain-soaked region of Galicia. Similarly, annual mean temperatures stretch from 2.6 °C in the northern Swedish town of Älvsbyn to 22.8 °C in Almonaster la Real, in southern Spain. These differences underscore the heterogeneous conditions under which climate actions are planned and implemented, making the database a rich foundation for comparative analysis (Table 2).

**Table 2:** Maximum and minimum values extracted from the database related to 4 features: Action plan budget, annual average precipitation, annual mean temperature and population density.

		Action plan budget (euro)	Annual average precipitation (mm)	Annual mean temperature ( °C)	Population density (capita/km^2^)
Maximum	City name	Paris	A BAÑA	Almonaster la Real	Paris
	Value	300,000,000	2923	22.8	20,623.1
Minimum	City name	Adamuz	Almáchar	Älvsbyn	Aldehuela de Liestos
	value	327.8	238	2.6	1.3

**Fig. 2:**
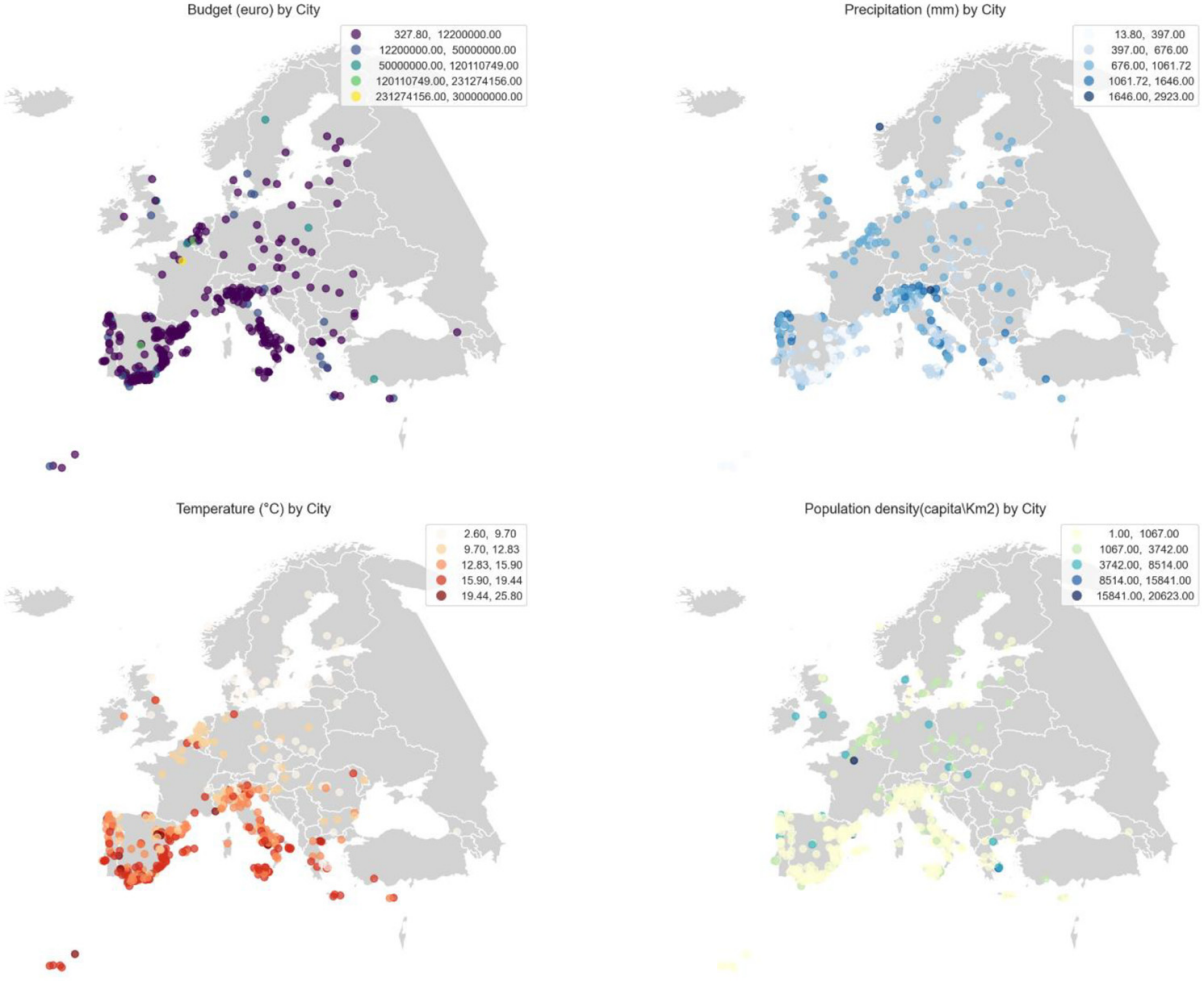
Spatial distribution of cities and key characteristics (budget, annual average precipitation, annual mean temperature and population density) in the climate action database.

As shown in Fig. 2, the analysis of the database shows some spatial patterns and potential correlations among some features like the four selected ones as example in the figure: Budget allocated to climate action plans, annual precipitation, mean annual temperature, and population density. Cities with higher allocated budgets tend to be concentrated in southern and western Europe regions that also experience higher average temperatures and greater population densities. This suggests a possible link between urban vulnerability to climate-related risks, such as heatwaves and urban density pressure, and the financial commitment to climate initiatives. These observations highlight how climatic and demographic pressures may influence urban investment in climate adaptation strategies.

## Limitations

Several limitations were encountered during data collection and curation. First, while 454 climate action plans were reviewed, inconsistencies in reporting formats across cities led to the exclusion of some data points, particularly for features like landfill area, which were missing in over 50 % of cases. Additionally, not all cities reported quantitative values for emissions reduction targets related to each climate action reported, budgets, or energy breakdowns, limiting the completeness of some action-related features. Second, spatial features derived from OpenStreetMap data may vary in accuracy depending on the city and country, especially for smaller municipalities where mapping detail is limited. Third, because the dataset is based on self-reported municipal plans, there may be inherent biases in how cities represent their climate actions, particularly in awareness strategies or qualitative goals.

Despite these limitations, careful preprocessing and imputation were applied to ensure the dataset remains suitable for machine learning applications.

## Ethics Statement

The authors confirm that the data collection and publication comply with the ethical requirements of *Data in Brief.* This work does not involve human subjects, animal experiments, or data collected from social media platforms.

## CRediT Author Statement

**Iskander Ben Rjiba:** Conceptualization, Methodology, Software, Validation, Formal analysis, Data Curation, Writing - Original Draft, Writing - Review \& Editing, Visualization. **Georgina Tóth-Nagy:** Conceptualization, Validation, Formal analysis, Investigation, Writing - Original Draft, Writing - Review \& Editing, Supervision. **Ágnes Rostási:** Formal analysis, Resources, Writing - Original Draft, Writing - Review \& Editing, Project administration, Funding acquisition. **Petra Gyurácz-Németh:** Conceptualization, Validation, Investigation, Data Curation, Writing - Original Draft, Writing - Review \& Editing. **Viktor Sebestyén:** Conceptualization, Methodology, Software, Validation, Formal analysis, Investigation, Resources, Data Curation, Writing - Original Draft, Writing - Review \& Editing, Visualization, Supervision.

## Acknowledgements

The research was funded by the Sustainable Development and Technologies National Programme of the Hungarian academy of Sciences (FFT NP FTA), and funding provided by the RRF-2.3.1–21–2022–00,014 National Multidisciplinary Laboratory for Climate Change. Viktor Sebestyén's contribution to this paper was supported by the János Bolyai Research Scholarship of the Hungarian Academy of Sciences (BO/00,889/24).

## Declaration of Competing Interest

The authors declare that they have no known competing financial interests or personal relationships that could have appeared to influence the work reported in this paper.

## Data Availability

githubArticle database (input).xlsx (Reference data)
